# CXCR4^+^ PD-L1^+^ neutrophils are increased in non-survived septic mice

**DOI:** 10.1016/j.isci.2025.112083

**Published:** 2025-02-22

**Authors:** Guilherme Cesar Martelossi Cebinelli, Maísa de Oliveira Leandro, Antonio Edson Rocha Oliveira, Kalil Alves de Lima, Paula Barbim Donate, Cleyson da Cruz Oliveira Barros, Anderson dos Santos Ramos, Victor Costa, Daniele Carvalho Bernardo Nascimento, Luis Eduardo Alves Damasceno, Amanda Curto Tavares, André Nicolau Aquime Gonçalves, Helder Takashi Imoto Nakaya, Thiago Mattar Cunha, José Carlos Alves-Filho, Fernando Queiroz Cunha

**Affiliations:** 1 Center for Research in Inflammatory Diseases (CRID), Department of Pharmacology, Ribeirao Preto Medical School – University of Sao Paulo (USP), Sao Paulo, SP, Brazil; 2 Department of Biochemistry and Immunology, Ribeirao Preto Medical School – University of Sao Paulo (USP), Sao Paulo, SP, Brazil; 3 Núcleo de Biologia Experimental, Universidade de Fortaleza (UNIFOR), Fortaleza, CE, Brazil; 4 Hospital Israelita Albert Einstein, São Paulo, SP, Brazil; 5 Department of Clinical and Toxicological Analyses, School of Pharmaceutical Sciences, University of São Paulo, São Paulo, Brazil

**Keywords:** Natural sciences, Biological sciences, Immunology, Immune response

## Abstract

The dysregulated host response to infections can lead to sepsis, a complex disease characterized by a spectrum of clinical phenotypes. Using scRNA-seq, we analyzed the immune cell of survived and non-survived CLP-septic mice to gain insights into the immunological mechanisms by which neutrophils contribute to the hyperinflammatory phenotype. Our findings reveal that non-survived mice exhibit increased frequencies of immature CXCR4^+^ PD-L1^+^ neutrophils in the bloodstream, accompanied by an accumulation of trafficking-specific CXCR4^+^ PD-L1^+^ neutrophils into the lungs. The IFN-gamma and LPS promote the PD-L1 expression on neutrophils and an activation profile associated with inflammation and organ damage. Notably, abrogating the IFN-gamma reduced susceptibility to CLP-sepsis and diminished CXCR4^+^ PD-L1^+^ neutrophils frequency. This study provides insights into the immune cell activation profiles associated with the worsening of the CLP-sepsis, and the CXCR4^+^ PD-L1^+^ neutrophils population highlighted here represents a promising target for therapeutic modulation in clinical sepsis hyperinflammatory phenotype.

## Highlights

• Non-survived septic mice exhibit increased apoptosis in T and B cells
• Non-survived septic mice exhibit a higher frequency of CXCR4+ PD-L1+ neutrophils
• Non-survived septic mice exhibit trafficking-specific PD-L1+ CXCR4+ neutrophils to the lungs
• IFNg promotes a neutrophils activation profile associated with inflammation and organ damage

## Graphical abstract

**Figure:**
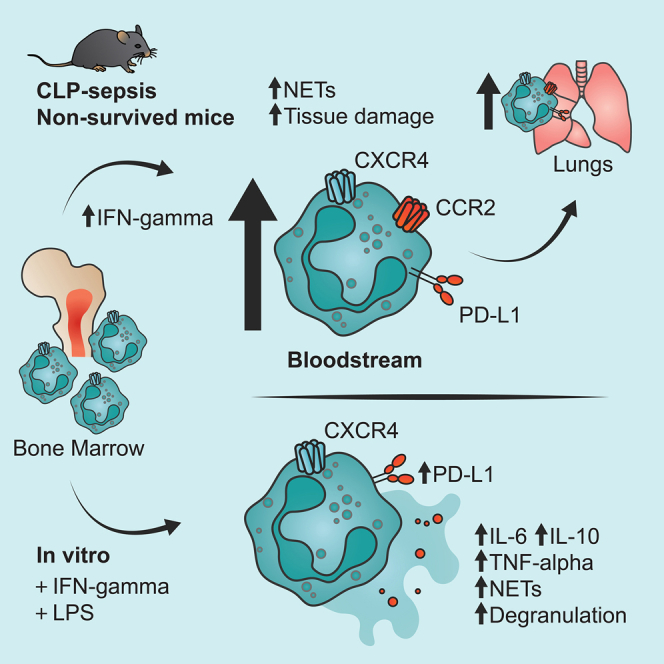

## Introduction

Sepsis is a critical condition characterized by a dysregulated host response to infections with life-threatening organ dysfunction.^1^ The pathogenesis of sepsis is initiated by the excessive activation of innate immune cells, epithelial cells, and endothelial cells through the recognition of diverse pathogen-associated molecular patterns (PAMPs) and damage-associated molecular patterns (DAMPs). The recognition of PAMPs and DAMPs induces complex intracellular signaling pathways involved in inflammation, adaptive immunity, and cellular metabolism. Although sepsis has been considered fundamentally mediated by the systemic inflammatory response, immunosuppression is also present from the beginning of the pathogenesis and it is an important factor for the disease pathogenesis.^2^ The progression of sepsis and outcome is determined by a complex balance between mechanisms responsible for controlling pathogen growth and mechanisms that avoid and repair organ dysfunctions caused by excessive inflammation.^3^ Recently, it was described that the pairwise combination of tumor necrosis factor (TNF) with IL-18, interferon-gamma (IFN-gamma), or IL-1beta recapitulates most of the cellular transcriptional signatures of sepsis across dysfunctional organs.^4^

The standardized treatment of sepsis is based on infection control, hemodynamic stabilization, and the modulation of the septic response. However, clinical trials targeting the well-known mediators associated with the response to sepsis, such as PAMPs, endotoxins, and inflammatory cytokines (e.g., IL-6, IFN-gamma, TNF, IL-1beta) have shown discrete or no efficient results.^2^^,^^4^ Gene-expression profiling of patients with sepsis has identified a spectrum of disease phenotypes that correlate with the host response and disease outcomes.^5^^,^^6^^,^^7^^,^^8^^,^^9^ The heterogeneity of responses and redundancy of biological pathways involved in this pathology probably explain the difficulty in implementing specific therapies for sepsis and contribute to its high worldwide mortality.^10^^,^^11^

The cecal ligation and puncture (CLP) sepsis model resembles the disease characteristics of the hyperinflammatory phenotype observed in patients with sepsis. This hyperinflammatory septic phenotype is the most dangerous, characterized by organ dysfunctions, septic shock, and higher mortality rates.^9^^,^^12^^,^^13^ The CLP induction in mice of the same inbred strain, weight, age, sex, and co-housed conditions evoked a heterogeneous host response, in which approximately half of the CLP-induced mice succumbed.^14^^,^^15^ In this model, neutrophils play dichotomous roles by promoting infection control while also contributing to organ damage.^16^ It has been recently described that PD-L1 upregulation through the IFN-gamma signaling pathway prolongs neutrophil cell survival and promotes lung injury.^17^ Moreover, neutrophils are capable of acquire an immunosuppressive profile and modulate the inflammatory response.^18^^,^^19^^,^^20^ Nevertheless, our current understanding of the cellular and molecular immune mechanisms underlying heterogeneous responses in sepsis remains limited. To address this gap, we take advantage of the CLP-septic survived and non-survived mice immune cell activation profiling using single-cell RNA sequencing, aiming to gain further insights into immunological mechanisms by which neutrophils contribute to the worsening of the hyperinflammatory septic phenotype and identify potential targets for the development of sepsis-specific therapeutics.

## Results

### A cohort of mice with identical genetic characteristics exhibits a heterogeneous host response to polymicrobial sepsis

**Figure 1:**
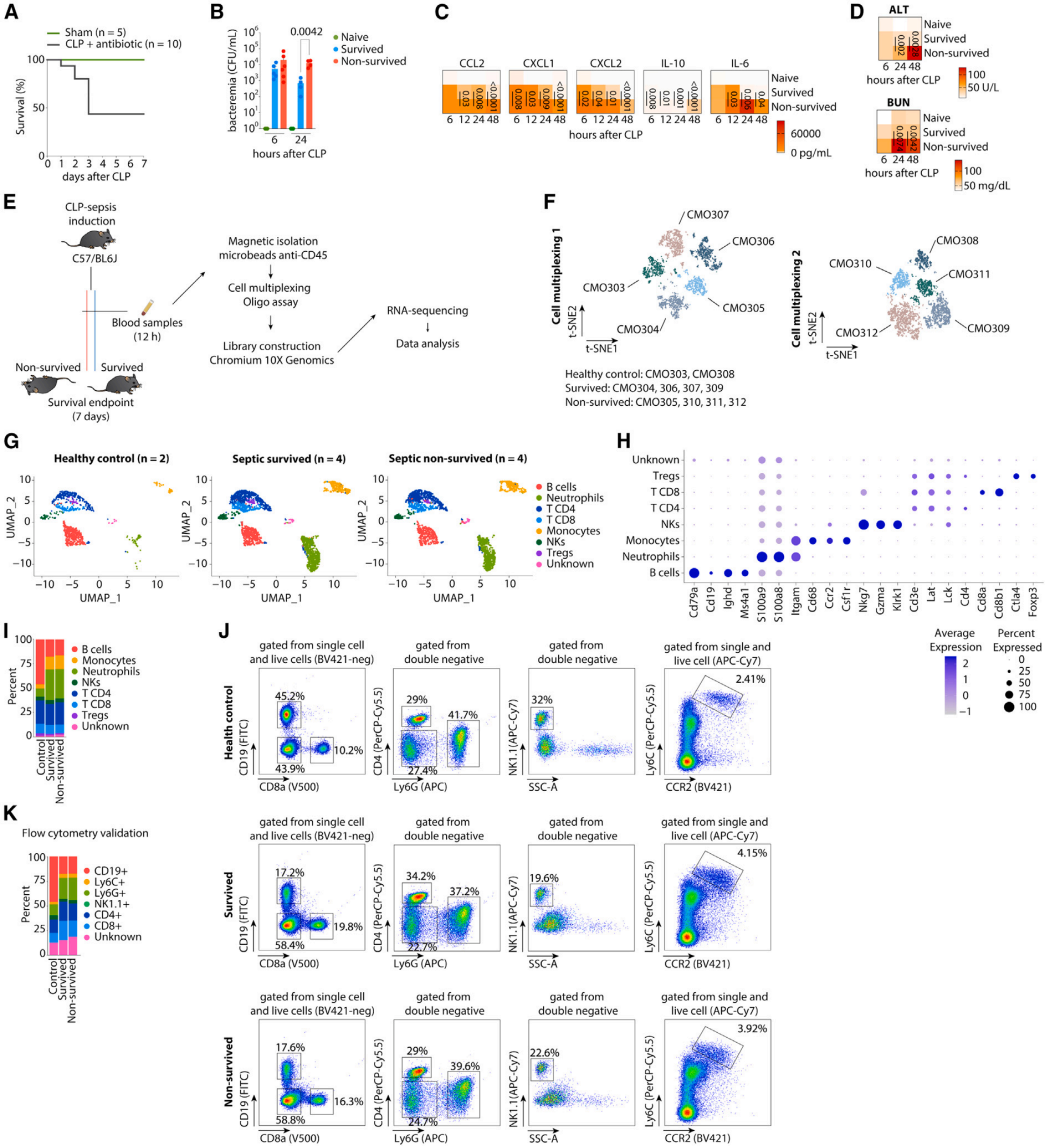
A cohort of mice with the same genetic characteristics exhibits a heterogeneous host response to polymicrobial sepsis (A) survival rate of homogeneous mice after the induction of CLP-sepsis followed by antibiotic treatment (*n* = 10). (B) Bacteremia (C) the plasmatic concentration of cytokines, and (D) the plasmatic concentration of alanine aminotransferase (ALT) and blood urea nitrogen (BUN) in control, survived, and non-survived mice after CLP-sepsis. (E) Schematic of the experimental approach used to perform single-cell RNA sequencing from magnetic isolated CD45^+^ leukocytes from the blood of survived (*n* = 4), non-survived (*n* = 5), and control (*n* = 2) mice after 12 h of CLP-sepsis induction. (F) Dimensionality reduction of the multiplexed samples (*n* = 10). (G) UMAP visualization of leukocytes isolated from controls, survived and non-survived after 12 h of CLP-sepsis induction, colored by each cluster. (H) Log-normalized expression per cell of genes used for the leukocyte population identification. (I) Percentage of leukocyte populations in controls, survived and non-survived CLP-septic mice. (J) Flow cytometry gating strategy used to identify the different subpopulations of leukocytes isolated from mice after 12 h of the CLP-sepsis induction. (K) Percentage of leukocyte populations observed in control, survived, and non-survived septic mice. The scRNA-seq data is from one experiment. The data are representative of two independent experiments with similar results. The data are shown as means or means ± s.e.m. Statistical significance was determined by one-way ANOVA followed by Tukey’s multiple comparisons test.

We observed that the induction of sepsis using the well-established cecal-ligation and puncture (CLP) model, followed by antibiotic treatment, in a group of mice from the same inbred strain with identical weight, age, sex, and co-housed, evoked a heterogeneous host response, as evidenced by the 7-day mortality rate of 56% (Figure 1A). In the follow-up analysis of blood samples, we did not observe differences in bacteremia, plasmatic concentration of liver and kidney damage markers, and plasmatic IL-6 concentrations assessed in the first 6 h (Figures 1B–1D), indicating a similar initial inflammatory response across all mice. However, we observed a sustained increase of bacteremia, plasmatic concentration of liver and kidney damage markers, and plasmatic IL-6 concentration in mice that did not survive after 24 and 48 h. In contrast, the survived mice showed a decrease in these parameters over time (Figures 1B–1D). Thus, our data suggest that, following the same initial inflammatory response, the non-survived mice exhibited a failure in infection resistance and tissue damage tolerance mechanisms.

Afterward, we took advantage of a single-cell RNA sequencing (scRNA-seq) technology to gain further insights into the cellular diversity and transcriptional landscape of the immunological heterogeneous response in mice during the initial phase of sepsis pathogenesis. For this, CD45^+^ cells were magnetically isolated from the blood of survived, non-survived, and control mice 12 h after CLP induction, multiplexed using molecular barcodes, and profiled using scRNA-seq (Figures 1E and 1F).

Uniform manifold approximation and projection (UMAP) for dimensionality reduction revealed clusters of leukocytes annotated as B cells, neutrophils, CD4 T cells, CD8 T cells, monocytes, NK cells, and Tregs (Figures 1G–1I). When assessing the frequency of the leukocyte populations identified in the scRNA-seq analysis and comparing them with frequency data from flow cytometry validation (Figures 1J and 1K), we observed similar frequency between survived and non-survived septic mice 12 h after CLP surgery. However, compared to the control group, septic mice exhibited a decreased frequency of B cells and an increased frequency of neutrophils and monocytes (Figures 1I–1K).

### Non-survived septic mice exhibit increased apoptosis in subpopulations of T and B cells

To explore the heterogeneous immune response to CLP-sepsis and gain further insights into hyperinflammatory sepsis phenotype, we next investigated the populations of each identified leukocyte in Figure 1 by performing additional dimensionality reduction to identify subpopulations and differences in the activation profiles between the outcome groups.

**Figure 2:**
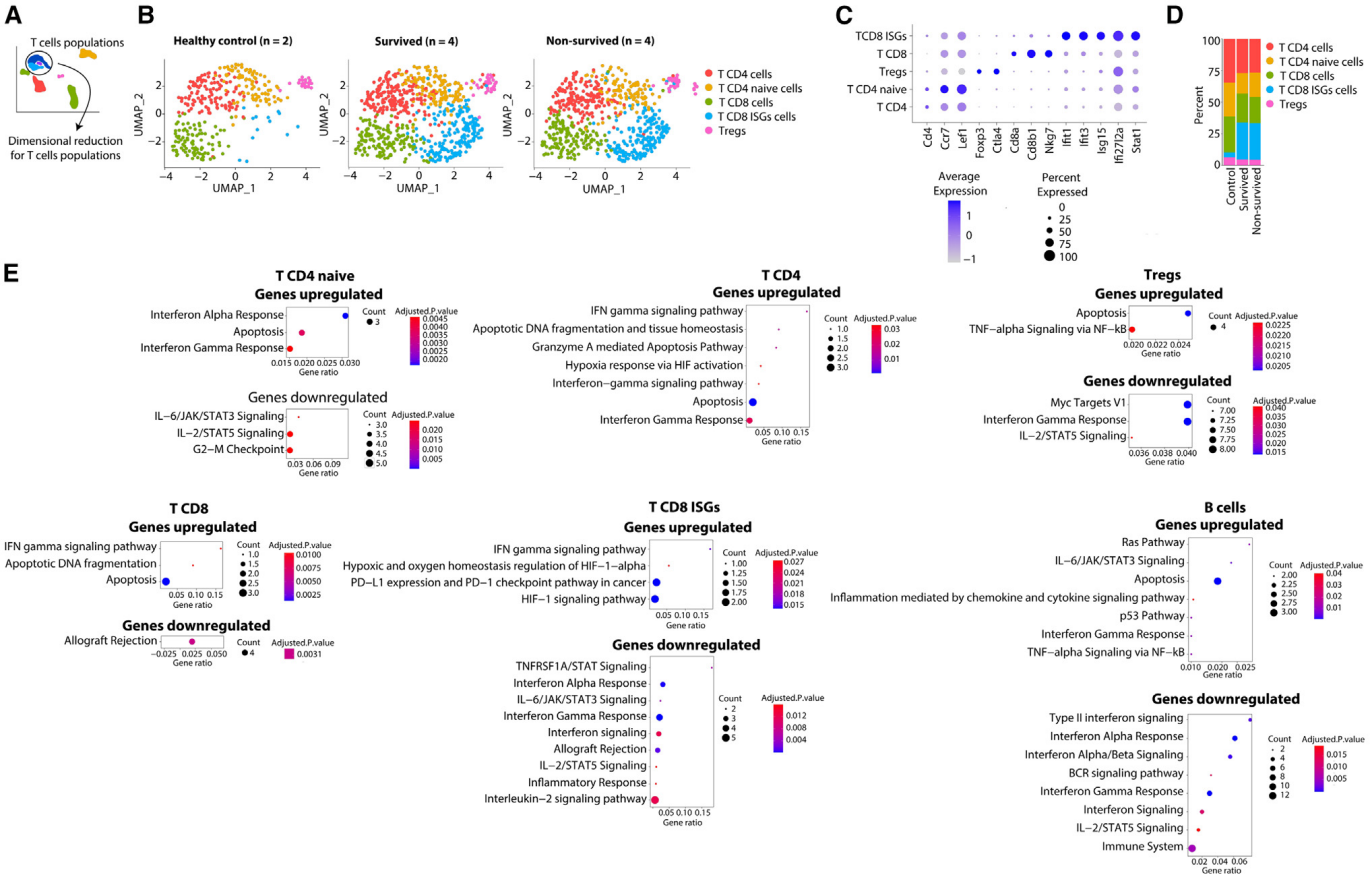
scRNA-seq identification of the B and T subpopulations activation signature of non-survived CLP-septic mice (A) Schematic of the dimensionality reduction analysis specific for T cell populations identified in Figure 1. (B) UMAP visualization of T cells isolated from controls (*n* = 2), survived (*n* = 4), and non-survived (*n* = 4) after 12 h of CLP-sepsis induction, colored by each cluster. (C) Log-normalized expression per cell of genes used for the T cells subpopulations identification. (D) Percentage of T cells subpopulations identified in control, survived, and non-survived septic mice. (E) Significantly enriched biological processes for the set of significantly upregulated and downregulated genes of the subpopulation of T and B cells in the non-survived septic mice compared with survived. The data are from one experiment. The data are shown as means. Statistical significance was determined by one-way ANOVA followed by Tukey’s multiple comparisons test.

A new dimensionality reduction analysis, grouping all T lymphocytes clusters identified in Figure 1, revealed subpopulations annotated by unsupervised gene expression markers as CD4^+^ T cells, CD4^+^ T naive cells, Tregs, CD8^+^ T cells, and CD8^+^ T with interferon-stimulated genes cells (ISGs) profile (Figures 2A–2D). Frequency analysis showed similar proportions of the identified populations between the survived and non-survived septic groups, and we observed a similar increase in the CD8^+^ T ISG cells in the septic groups compared to the control group (Figure 2D).

Next, we performed gene enrichment analysis for biological processes in the set of differentially expressed genes (DEGs) from each lymphocyte subpopulation between survived and non-survived groups (Tables S1–S6). The analysis revealed an enrichment of upregulated gene expression related to apoptosis in all T cell populations, except in the CD8^+^ T ISGs, from the non-survived septic group in comparison with the survived group (Figure 2E). CD4^+^ T naive, CD4^+^ T, CD8^+^ T, and CD8^+^ T ISGs from the non-survived group showed an enrichment of genes related to the IFN-gamma signaling pathway, while CD4^+^ T and CD8^+^ T ISGs displayed enrichment in HIF-1alpha signaling pathway (Figure 2E). Additionally, in the CD8^+^ T ISGs, we found an enrichment of the PD-L1 expression signaling pathway (Figure 2E).

Moreover, we did not observe distinct subpopulations of B cells after a new dimensionality reduction analysis of our data, possibly due to the low number of cells analyzed. However, the enrichment analysis for biological processes revealed an increased gene expression related to the apoptosis pathway and decreased genes related to the BCR signaling in the B cell population in the non-survived group compared to the survived group (Figure 2E).

Thus, our findings demonstrated an enrichment of the apoptosis pathway in the subsets of T and B cells, but not in the CD8^+^ T ISGs population in the non-survived group. The CD8^+^ T ISGs population exhibited an immunosuppressive profile, as we observed an enrichment of genes related to the HIF-1alpha and PD-L1/PD-1 expression pathways.

### Non-survived septic mice exhibit an increased frequency of immature monocytes with an immunosuppressive activation profile

**Figure 3:**
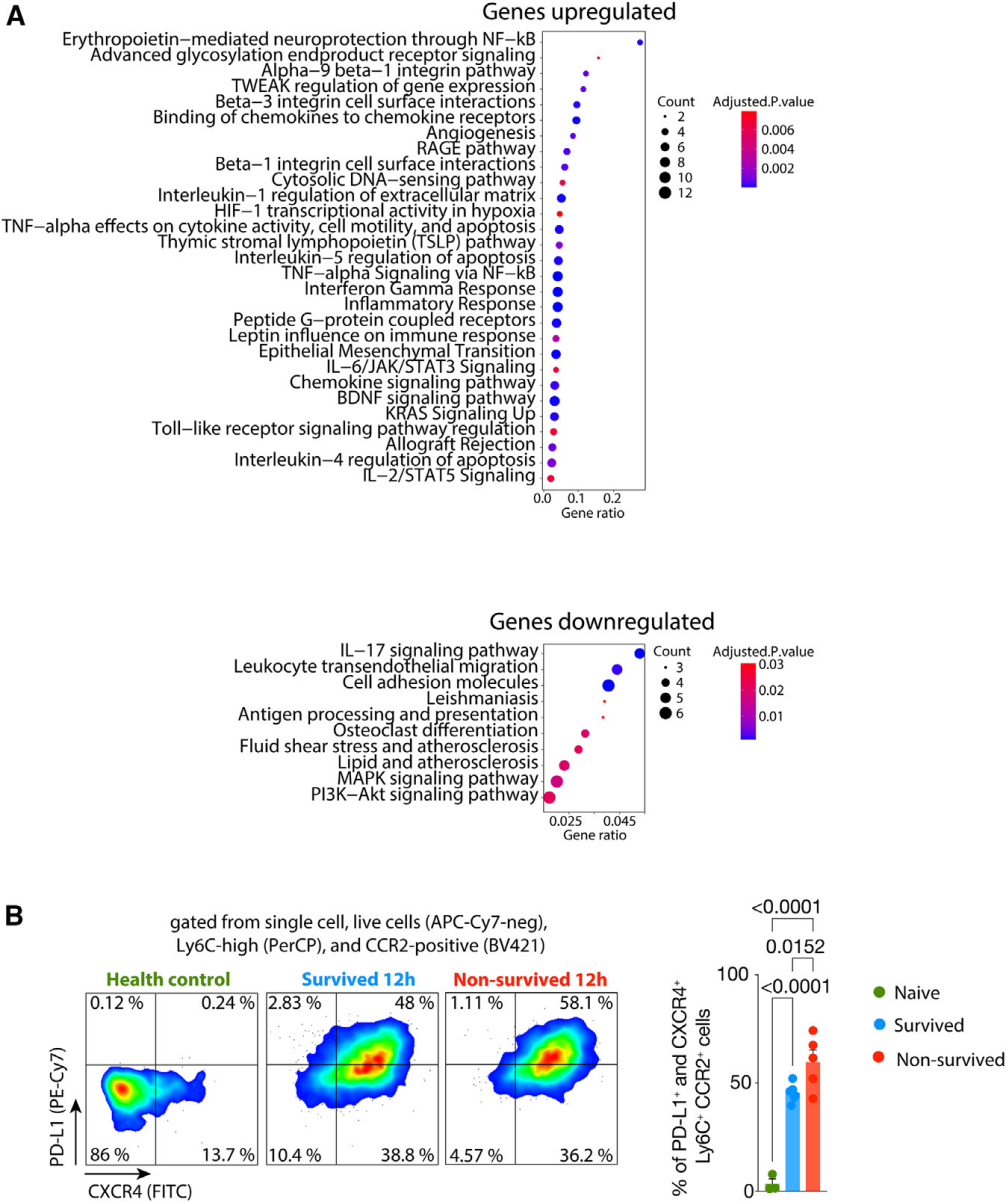
The activation signature of monocytes related to the CLP-sepsis outcome (A) Significantly enriched biological processes for the set of significantly upregulated and downregulated genes of the monocytes identified in Figure 1 in the non-survived septic mice compared with survived. (B) (left) Flow cytometry gating strategy to identify populations of immature monocytes isolated from mice after 12 h of the CLP-sepsis induction, and (right) the frequency of PD-L1+ CXCR4+ monocytes observed in controls (*n* = 3), survived (*n* = 5) and non-survived septic mice (*n* = 5). The scRNA-seq data is from one experiment. The flow cytometry data is representative of two independent experiments with similar results. IThe data are shown as means ± s.e.m, and each data point represents one mouse. Statistical significance was determined by one-way ANOVA followed by Tukey’s multiple comparisons test.

Monocytes play a protective role in mediating host resistance to infections and a deleterious role in reducing host tolerance through pro-inflammatory mechanisms in sepsis.^15^^,^^21^ The gene enrichment analysis for biological processes in the set of DEGs of monocytes from the comparison of survived and non-survived groups (Tables S7 and S8) revealed enrichment of genes related to the TNF-alpha, IL-6, IL-1, IFN-gamma, and HIF-1 signaling pathways, along with a decrease in pathways related to cell migration and antigen processing and presentation in the monocytes from non-survived septic mice (Figure 3A). In fact, it has been reported that monocytes undergo functional reprograming during sepsis, transitioning from a pro-inflammatory to an immunosuppressive state, a process regulated by the HIF-1alpha signaling pathway (Shalova et al., 2015).

The IFN-gamma signaling pathways induce HIF-1alfa expression or stabilization in macrophages and promote PD-L1 expression in macrophages and neutrophils.^17^^,^^22^ Our flow cytometry data analysis revealed an increased frequency of PD-L1^+^ CXCR4^+^ monocytes in the blood of the non-survived septic group compared with survived septic mice (Figure 3B). The CXCR4 expression retains a monocyte precursor in the bone marrow, which replenishes the peripheral mature monocyte pool during infections (Chong et al., 2016). These findings suggest an increase in developmentally immature monocytes with an immunosuppressive activation profile in the non-survived septic group.

### Non-survived septic mice exhibit an increased frequency of immature and CXCR4^+^ PD-L1^+^ neutrophils

**Figure 4:**
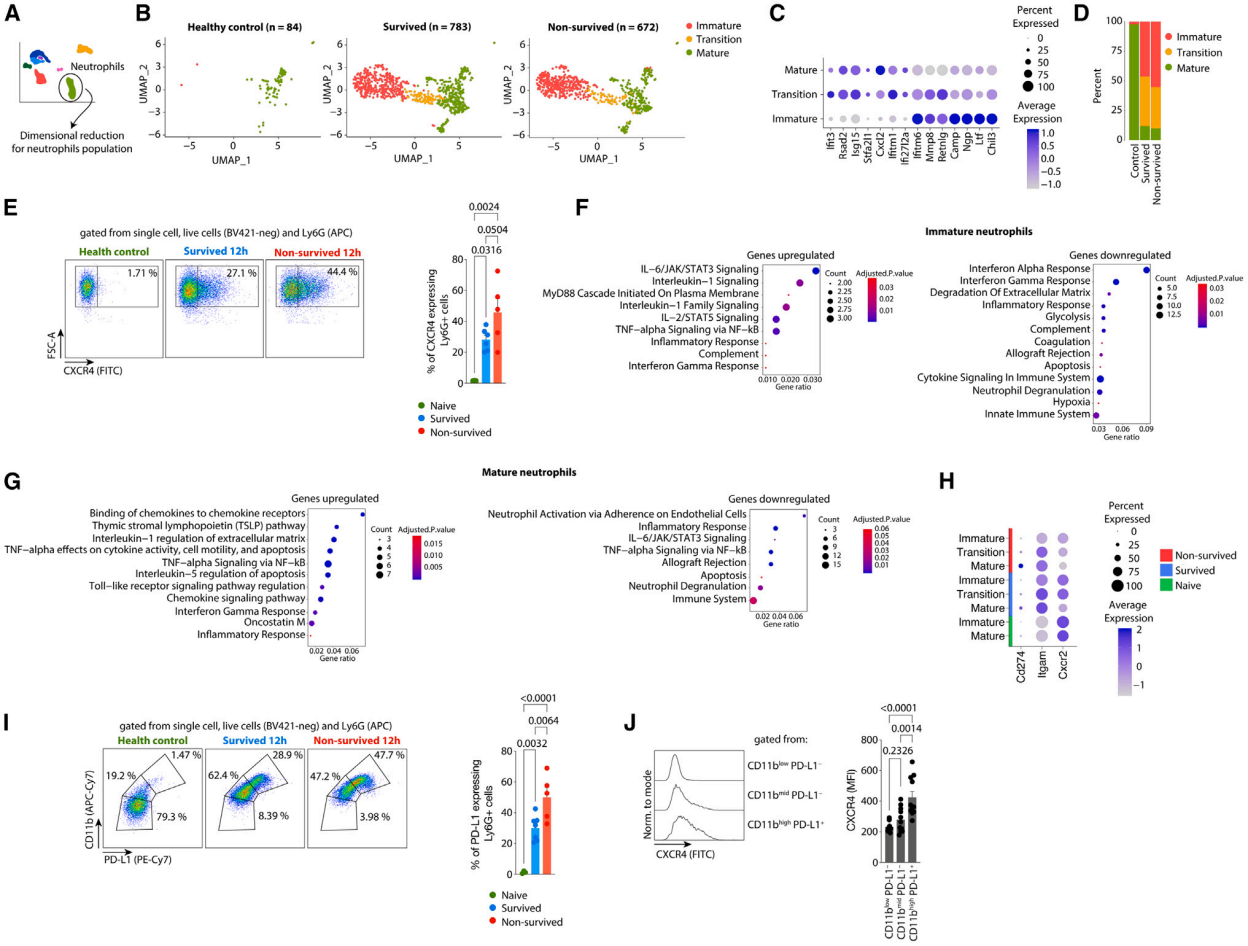
The CXCR4+ PD-L1+ neutrophil subpopulation is increased in non-survived septic mice (A) Schematic of the dimensionality reduction analysis specific for neutrophils identified in Figure 1. (B) UMAP visualization of neutrophils isolated from controls (*n* = 2), survived (*n* = 4), and non-survived (*n* = 4) after 12 h of CLP-sepsis induction, colored by each cluster. (C) Log-normalized expression per cell of genes used for the neutrophils subpopulations identification. (D) Percentage of neutrophils subpopulations identified in control, survived, and non-survived septic mice. (E) (left) flow cytometry gating strategy to identify the population of CXCR4^+^ neutrophils from the blood of mice after 12 h of the CLP induction and (right) the percentage of CXCR4^+^ neutrophils populations observed in control (*n* = 3), survived (*n* = 6), and non-survived (*n* = 5) CLP-septic mice. (F and G) Significantly enriched biological processes for the set of significantly upregulated and downregulated genes of the subpopulation of neutrophils in the non-survived septic mice compared with survived. (H) Expression of selected genes in the populations of neutrophils identified in Figure 4B. (I) (left) flow cytometry gating strategy to identify the population of PD-L1^+^ neutrophils from the blood of mice after 12 h of the CLP induction and (right) the percentage of PD-L1^+^ neutrophils populations observed in control (*n* = 3), survived (*n* = 7), and non-survived (*n* = 5) CLP-septic mice. (J) Expression of CXCR4 in the subpopulations of neutrophils identified in Figure 4I. The scRNA-seq data is from one experiment. The flow cytometry data is representative of two independent experiments with similar results. The data are shown as means ± s.e.m, and each data point represents one mouse. Statistical significance was determined by one-way ANOVA followed by Tukey’s multiple comparisons test.

Neutrophils play both protective and deleterious roles in experimental models of sepsis, promoting infection control while also contributing to organ damage.^16^ In severe cases of clinical sepsis, a “left shift” in leukocyte counts and an increase in both immature and mature neutrophils with an immunosuppressive profile are observed.^19^ The dimensionality reduction specifically applied to the neutrophils cluster, combined with annotation based on developmental gene expression markers^23^ revealed populations of mature, transitional, and immature neutrophils in our experimental groups (Figures 4A–4D). The frequency analysis showed that sepsis induction leads to increased frequencies of transition and immature neutrophil populations compared to the control group. Additionally, we observed a higher frequency of immature neutrophils in the non-survived group compared to the survived group (Figure 4D).

CXCR4 expression plays a critical role in retaining immature neutrophils in the bone marrow. Upon maturation, the emigration of mature neutrophils from bone marrow to the bloodstream is regulated by the downregulation of CXCR4 and upregulation of CXCR2.^24^^,^^25^^,^^26^^,^^27^ In circulation, aged neutrophils upregulate the CXCR4 expression, trafficking their back to the bone marrow for elimination.^27^ However, the CXCR4 expression also characterizes the immature population of neutrophils.^20^ Notably, we observed an increased frequency of CXCR4-expressing neutrophils in the non-survived septic mice compared to survived mice (Figure 4E). This finding aligns with the scRNA-seq data (Figure 4D), which showed an increased frequency of immature neutrophils in the non-survived compared to survived.

Next, we performed gene enrichment analysis for biological processes in the set of DEGs generated by comparing neutrophil populations from survived and non-survived groups (Table S9). Our results demonstrated an upregulation of DEGs related to the IL-6, IL-1, IL-2, and TNF-alpha signaling pathways, along with a downregulation of DEGs related to the apoptosis, neutrophils degranulation, glycolysis, and IFN-alpha and IFN-gamma response in immature neutrophils from non-survived septic mice (Figure 4F). In mature neutrophils from non-survived septic mice, we observed an upregulation of DEGs related to TNF-alpha, IFN-gamma, and Toll-like signaling pathway and downregulation of DEGs related to apoptosis, IL-6 signaling, and neutrophil degranulation (Figure 4G).

The IFN-gamma and LPS signaling pathways induce PD-L1 expression in macrophages and neutrophils.^17^ Consistently, gene enrichment analysis for biological processes demonstrated an upregulation of IFN-gamma and Toll-like receptor signaling pathways, accompanied by the increased expression of *Cd274* (PD-L1 gene) in the mature neutrophil population from non-survived septic mouse (Figures 4G and 4H, respectively). The flow cytometry analysis revealed an increased frequency of the PD-L1 expressing neutrophils in non-survived mice (Figure 4I). The PD-L1^+^ neutrophils exhibited higher levels of CD11b, indicating that these cells are more activated,^28^ and also showed increased expression of CXCR4 (Figures 4J and 6I). Taken together, these results indicated that non-survived septic mice exhibit an increased frequency of immature CXCR4^+^ PD-L1^+^ neutrophils.

### Non-survived septic mice exhibit an increased frequency of CXCR4^+^ PD-L1^+^ neutrophils in the lungs

Considering that monocyte and other PDL-1-expressing cells play immunosuppressive role^29^ and that neutrophils can also exhibit an immunosuppressive profile and modulate the inflammatory response,^18^^,^^19^^,^^20^ we further investigated the population of CXCR4^+^ PD-L1^+^ neutrophils in our septic model.

The CXCR4 expression plays an important role in neutrophil marginalization within the lungs microvasculature.^24^ In experimental sepsis models, neutrophils in the pulmonary tissue show increased CXCR4 expression, which promotes their retention in the lungs.^30^^,^^31^

Next, we take advantage of IL-6 concentration for mortality prediction to define groups of predicted to survive and predicted to non-survive, enabling access to invasive biological specimens as organs and peritoneal lavage. The mortality prediction was performed as previously described by Remick and collaborators.^14^ A receiver operating characteristic (ROC) analysis was conducted using IL-6 concentrations at 24 h after CLP-sepsis induction and the outcome was assessed until 7 days. The results showed that plasmatic IL-6 concentrations higher than 4165 pg/mL had 100% specificity for identifying non-survived mice, while concentrations lower than 2790 pg/mL had 100% specificity for identifying survived mice (Figure S1A–S1B).

**Figure 5:**
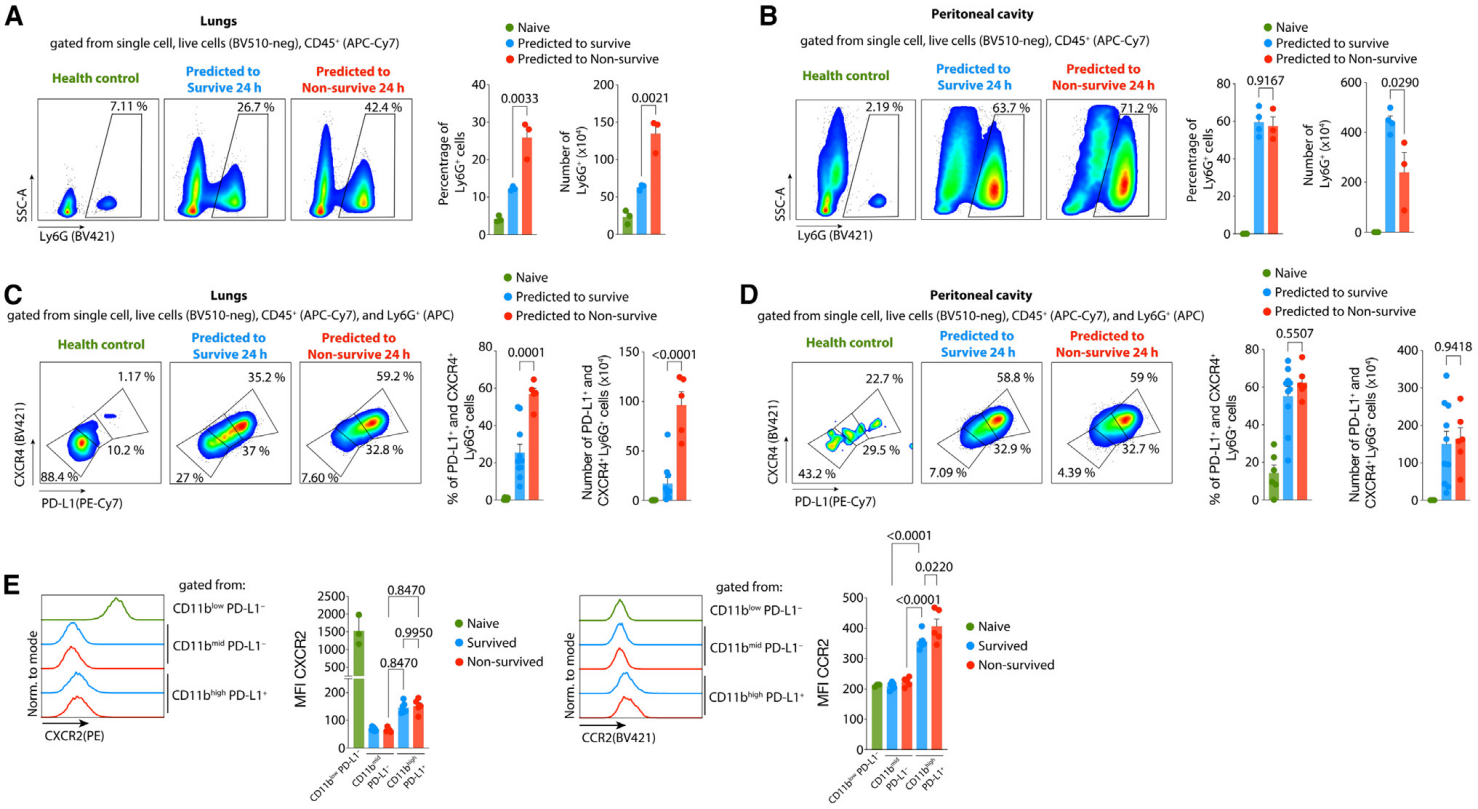
Non-survived septic mice exhibit an increased accumulation of neutrophils PD-L1+ CXCR4+ in the lungs (A and B) (left) Flow cytometry gating strategy to identify neutrophils (Ly6G^+^) and (right) percentage and the number of neutrophils from A, lungs and B, peritoneal cavity after 12 h of the CLP induction in controls, predicted to survive, and predicted to non-survive CLP-septic mice. (C and D) (left) Flow cytometry gating strategy to identify neutrophils (Ly6G^+^) and (right) percentage and number of PD-L1^+^ CXCR4^+^ neutrophils from C, lungs and D, peritoneal cavity after 12 h of the CLP induction in controls, predicted to survive, and predicted to non-survive CLP-septic mice. (E) Expression of CXCR2 and CCR2 in the subpopulations of neutrophils identified in Figure 4I. The data is representative of two independent experiments with similar results. The data are shown as means ± s.e.m, and each data point represents one mouse. Statistical significance was determined by one-way ANOVA followed by Tukey’s multiple comparisons test.

Using the mortality prediction cohort, we observed an increased bacterial load in the infection focus (Figure S1C). In the lungs, kidneys, heart, and liver, the concentration of inflammatory cytokines and chemokines was higher in predicted to non-survive mice compared to predicted to survive mice 24 h after CLP induction (Figure S1 1day-e). Additionally, we observed an increased number of neutrophils in the lungs and a decreased number of these cells in the peritoneal cavity of mice predicted to not survive from sepsis in comparison to those predicted to survive (Figures 5A and 5B). Given the results demonstrating the increased PD-L1^+^ CXCR4^+^ neutrophils frequency in the blood of non-survived mice, we next analyzed the distribution of these cells in the infection focus and lungs of mice predicted to survive and predicted to non-survive. While the number of CXCR4^+^ PD-L1^+^ neutrophils in the peritoneal cavity was similar between the predicted to survive and to non-survive (Figure 5D), we observed an increased number of CXCR4^+^ PD-L1^+^ neutrophils in the lungs of mice predicted to non-survive (Figure 5C).

In mice and patients with severe sepsis, the expression of the neutrophil canonical chemokine receptor CXCR2 has been shown to internalize in circulating neutrophils, which is associated with the impaired migration of these cells to the infection focus.^32^^,^^33^^,^^34^ Additionally, it has been demonstrated that CCR2 is upregulated in neutrophils from septic mice and patients, mediating their infiltration into organs such as lungs, kidneys, and heart.^35^ In our flow cytometry analysis, we observed a similar decrease in CXCR2 expression on both PD-L1^-^ and PD-L1^+^ neutrophils in the bloodstream of both survived and non-survived mice (Figure 5E). However, CCR2 expression was increased only on PD-L1^+^ neutrophils in both survived and non-survived mice (Figure 5E).

Taken together, non-survived septic mice exhibit an increased accumulation of trafficking-specific neutrophils PD-L1^+^ CXCR4^+^ in the lungs compared to survived septic mice, which may result from the CXCR4 expression and upregulation of CCR2. The PD-L1^+^ expression has been described to promote a prolongated survival in neutrophils, which is associated an increased lung injury capacity.^17^^,^^31^ This could be associated with the heightened intensity of organ damage and increased susceptibility in the non-survived septic mice.

### Interferon-gamma-gamma induces PD-L1 expression on neutrophils and modulates the experimental sepsis susceptibility

**Figure 6:**
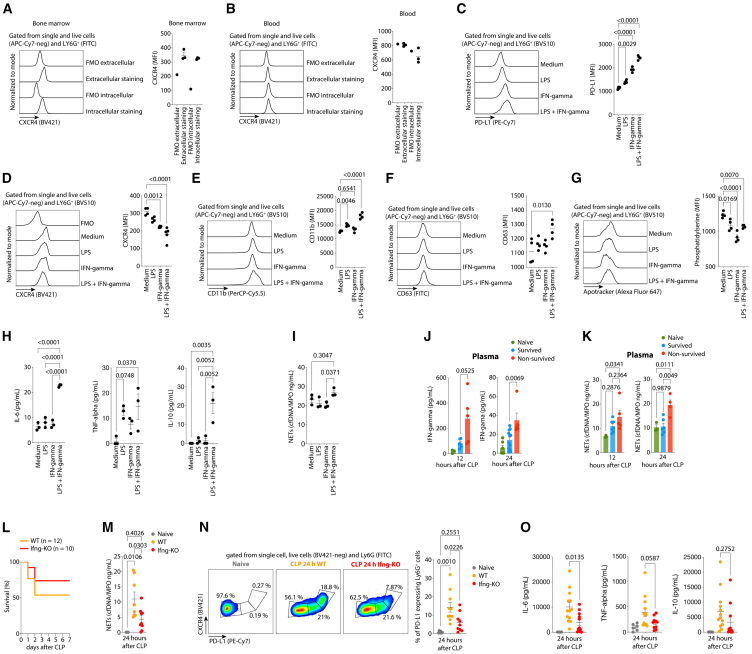
IFN-gamma induces PD-L1 expression on neutrophils and increases the susceptibility of mice to the CLP-sepsis (left) flow cytometry representation, and (right) the median of fluorescence intensity (MFI) of CXCR4 on (A), neutrophils from bone marrow, and (B) neutrophils from blood. (left) flow cytometry representation, and (right) the median of fluorescence intensity (MFI) of (C), PD-L1, (D) CXCR4, (E) CD11b, (F) CD63, (G) Phosphatidylserine in CXCR4^+^ neutrophils isolated from bone marrow after 4 h of stimulation with LPS and IFN-gamma. Evaluation of (H), IL-6, TNF-alpha, and IL-10 concentration, and (I), NETs concentration in CXCR4^+^ neutrophils isolated from bone marrow after 4 h of stimulation with LPS and IFN-gamma. Evaluation of (J), IFN-gamma concentration, and (K) NETs concentration in plasma of controls, survived and non-survived mice after 24 h of CLP-sepsis. (L) Survival rate of WT and IFN-gamma deficient mice after the induction of CLP-sepsis (*n* = 22). (M) Plasmatic concentration of NETs in WT and IFN-gamma deficient mice after 24 h of CLP-sepsis. (N) (left) flow cytometry gating strategy, and (right) the frequency of the PD-L1^+^ neutrophils after 24 h of CLP-sepsis induction in WT and IFN-gamma deficient mice. (O) Plasmatic concentration of IL-6, TNF-alpha, and IL-10 in WT and IFN-gamma deficient mice after 24 h of CLP-sepsis. The data are representative of two independent experiments with similar results. The data are shown as means ± s.e.m, and each data point represents one mouse. Statistical significance was determined by one-way ANOVA followed by Tukey’s multiple comparisons test.

The capacity of IFN-gamma and LPS signaling pathways to induce PD-L1 expression in macrophages and neutrophils has been previously described.^17^^,^^22^ Our findings demonstrate that neutrophils express CXCR4 exclusively in the bone marrow and not in the bloodstream under homeostatic conditions (Figures 6A and 6B), consistent with prior studies.^24^^,^^25^^,^^26^^,^^27^ The CXCR4^+^ neutrophils isolated from bone marrow can upregulate the PD-L1 expression upon IFN-gamma or LPS stimulation (Figure 6C). The surface expression of CXCR4 on neutrophils decreased after 4 h of cell culture within IFN-gamma or LPS (Figure 6D). Additionally, these stimuli increased the expression of CD11b and CD63, indicating enhanced neutrophil activation and increased granule release, respectively (Figures 6E and 6F). We also observed a downregulation of Annexin V staining, suggesting that IFN-gamma and LPS enhance neutrophil survival (Figure 6G). These stimuli were also responsible for upregulating the release of IL-6, TNF-alpha, IL-10, and NETs by CXCR4^+^ PD-L1^+^ neutrophils (Figures 6H and 6I).

*In vivo*, the plasmatic concentrations of IFN-gamma were higher in non-survived mice compared to survived mice (Figure 6J), coinciding with the increased frequency of CXCR4^+^ PD-L1^+^ neutrophils (Figures 4I and 4J). The IFN-gamma signaling pathway has been shown to increase of NET release and prolong neutrophil survival.^17^^,^^36^^,^^37^ In line with this, our gene enrichment analysis for biological processes revealed a downregulation of the genes related to apoptosis in both immature and mature neutrophils from non-survived mice (Figures 4F and 4G). Additionally, non-survived septic mice exhibited higher plasmatic concentrations of NETs compared to survived septic mice (Figure 6K). Supporting these findings, IFN-gamma deficient mice showed a 50% reduction in susceptibility to sepsis, a lower plasmatic concentration of NETs, IL-6, and reduced frequency of CXR4^+^ PD-L1^+^ neutrophil compared to WT mice under CLP-sepsis (Figures 6J–6l).

Together, these findings demonstrate an activation profile of CXCR4^+^ PD-L1^+^ neutrophils, exhibiting a phenotype associated with inflammation and organ damage, potentially impacting survival outcomes in the CLP-sepsis model.

## Discussion

The sepsis progression is highly heterogeneous due to patient individuality, pathophysiological variations, comorbidities, and distinct immunological responses.^38^ Understanding the underlying disease phenotypes is crucial for developing personalized therapeutic approaches.^39^ In our study, we provide molecular and functional insights into the immune cell activation profile associated with the heterogeneous responses observed in CLP-sepsis. This model resembles the hyperinflammatory sepsis phenotype, characterized by higher levels of inflammatory mediators, organ dysfunctions, septic shock, and increased mortality rates.^9^ Our findings highlight mechanisms that may contribute to the exacerbation of the hyperinflammatory sepsis phenotype and provide valuable therapeutic insights for managing this specific clinical phenotype.

In this study, using an unbiased experimental approach, we transcriptomic profiled leukocytes from both survived and non-survived septic mice. Our findings revealed an enrichment of apoptotic signaling pathway in the subsets of T and B cells from the non-survived septic group, but not in the CD8^+^ T ISGs population. The apoptosis of B and CD4^+^ T cells, as well as immunosuppression, have been previously associated with worst outcome in patients with sepsis.^11^^,^^40^^,^^41^ The role of monocytes in sepsis is widely described as orchestrating the host immune response, releasing inflammatory cytokines or transitioning to an immunosuppressive activation profile.^42^^,^^43^ In line with this, we observed a transitional transcriptional signature in monocytes from a pro-inflammatory to an immunosuppressive state in the non-survived septic mice. This process, regulated by the expression of HIF-1alpha, has been previously described in patients with sepsis.^42^ Additionally, we observed increased expression of PD-L1 in the monocytes from non-survived mice, which was been previously associated with CLP-sepsis-induced lymphocytes depletion and higher mortality in septic patient (Hotchkiss et al., 2013; Shao et al., 2016; Y. Zhang et al., 2010). Neutrophils play paradoxical roles in sepsis, both promoting infection control and contributing to organ damage.^16^ Complicating this further, neutrophils have also been described to exhibit an immunosuppressive profile, modulating the inflammatory response and promoting contact-dependent apoptosis of T and B cells.^18^^,^^19^^,^^20^^,^^44^ In this context, the increased frequency of CXCR4^+^ PD-L1^+^ neutrophils and also monocytes may be correlated with the enriched apoptosis pathways of the B and T cells in the non-survived mice.

The increased frequency of immature CXCR4^+^ PD-L1^+^ neutrophils in the bloodstream of non-survived mice is followed by an increased accumulation of trafficking-specific CXCR4^+^ PD-L1^+^ neutrophils in the lungs. The excessive TLR4 signaling promotes a failure of neutrophil recruitment to infected tissues due to CXCR2 internalization during sepsis. Concomitantly, these neutrophils increased their recruitment to tissues through CCR2 expression.^35^^,^^45^ In our model, the CXCR4^+^ PD-L1^+^ neutrophils in the bloodstream exhibited increased CCR2 expression. In addition to CXCR4, it can enhance their migration to the lungs, contributing to tissue damage.^30^^,^^31^ On the other hand, the impairment migration of neutrophils to the infection focus and decreased bacterial clearance may explain the increased bacterial load observed in the non-survived septic mice. Consistent with our findings, CXCR4^+^ immature neutrophils, also described as low-density neutrophils, which have an immunosuppressive profile, are increased in patients with sepsis. These neutrophils exhibit reduced apoptosis and impaired bacterial clearance.^18^^,^^19^^,^^20^ Furthermore, the PD-L1^+^ neutrophils accumulate in the lungs after a thermal injury, and they are effective at phagocytosing bacteria but exhibit reduced bacterial killing capacity.^46^ Additionally, PD-L1^+^ neutrophils are characterized by delayed apoptosis and contribute to lung injury, increasing the mortality in CLP-sepsis models.^17^^,^^31^ Our *in vitro* experiments, demonstrated that IFN-gamma and LPS increased PD-L1 expression on CXCR4^+^ neutrophils from bone marrow, and also promoted activation, granule, IL-6, and NETs release and less apoptosis markers. Therefore, the increased presence of PD-L1^+^ CXCR4^+^ neutrophils in the lungs correlates with the worsening phenotype in the non-survived septic group. When we blocked the IFN-gamma signaling pathway using IFN-gamma deficient mice, we observed reduced susceptibility to CLP-sepsis and less CXCR4^+^ PD-L1+ neutrophils frequency. In addition, the IFN-gamma signaling pathway also contributes to the release of NETs release and prolongs of neutrophil survival,^17^^,^^36^^,^^37^ both of which have been associated with increased tissue damage in sepsis.^17^^,^^31^^,^^47^

Furthermore, the increased plasmatic concentration of IFN-gamma in non-survived septic mice correlates with the increased frequency of CXCR4^+^ PD-L1^+^ neutrophils, which may explain the heightened organ damage, higher NETs concentration, and B and T apoptosis in these group of mice, further supporting the association between CXCR4^+^ PD-L1^+^ neutrophils and the increased susceptibility in non-survived septic mice. However, this may create a feedback loop, as the upregulated production of IFN-gamma or a higher bacterial load (LPS) can increase the frequency of CXCR4^+^ PD-L1^+^ neutrophils, further amplifying the hyperinflammatory state in sepsis. Thus, the increased levels of IFN-gamma in non-survived mice appear to modulate the neutrophils profile, potentially playing a role in the heterogeneous immune response observed among genetically similar mice during sepsis.

Collectively, our work provides molecular and functional insights into the immune cell activation profile associated with the worsening of a septic hyperinflammatory phenotype in an experimental model. The CXCR4^+^ PD-L1^+^ neutrophils population and elevated plasmatic IFN-gamma levels highlighted here present potential targets for therapeutic modulation in the clinical sepsis hyperinflammatory phenotype.

### Limitations of the study

The limitations of this study include the incomplete exploration of the effector mechanisms by which CXCR4^+^ PD-L1^+^ neutrophils and monocytes contribute to the worsening of CLP-sepsis. Further research is needed to investigate the effector mechanisms of PD-L1^+^ CXCR4^+^ neutrophils and monocytes underlying lymphocyte apoptosis and tissue damage in non-survived mice. Additionally, the highlighted targets must be modulated to assess their therapeutic potential, and our findings should be translated to confirm their specificity in the hyperinflammatory phenotype observed in clinical sepsis. The experiments were conducted exclusively in male mice; therefore, the results have limitations in terms of gender generalizability.

## Resource availability

### Lead contact

Further information and resource requests should be directed to the lead contacts, Guilherme Cesar Martelossi Cebinelli (cebinelli.gcm@gmail.com) and Fernando de Queiroz Cunha (fdqcunha@fmrp.usp.br), who will fulfill them.

### Materials availability

This study did not generate new unique reagents.

### Data and code availability

• Single-cell RNA-seq data have been deposited at GEO and are publicly available as of the date of publication. The raw and processed data can be accessed in the GEO database under the accession number GEO: GSE287865.
• This article does not report the original code.
• Any additional information required to reanalyze the data reported in this article is available upon request from the lead contact.

## Acknowledgments

We would like to thank all members of the Inflammation and Pain Laboratory and Center of Research on Inflammatory Diseases (CRID) for their valuable technical support and insightful discussions throughout this work. We also express our gratitude to the Flow Cytometry Core Facility and the Graduate Program in Basic and Applied Immunology for their technical support and contributions to the academic development. This work was supported by a scholarship and grant from the Fundação de Amparo a Pesquisa do Estado de São Paulo (FAPESP), under grant numbers 2019/15070-0 and 2013/08216-2.

## Author contributions

G.C.M.C. designed and performed the experiments, analyzed and interpreted the data and wrote the article. A.E.R.O. and A.N.A.G. analyzed the scRNA-Seq data and provided intellectual contributions. M.d.O.L. and C.d.C.O.B. helped with the neutrophils cell culture experiments and provided intellectual contributions. K.A.d.L. and P.B.D helped with the flow cytometry experiments and provided intellectual contributions. D.C.N and L.E.A.D helped with the scRNA- 8Seq experiments and provided intellectual contributions. A.d.S.R, V.C., and A.C.T assisted with the experimental procedures. H.T.I.N., T.M.C., J.C.A.F., and F.d.Q.C provided intellectual contribution, oversaw data analysis and interpretation, and wrote the article.

## Declaration of interests

The authors declare no conflicts of interest.

## Declaration of generative AI and AI-assisted technologies in the writing process

During the preparation of this work the author(s) used ChatGPT in order to improve language and readability. After using this tool/service, the author(s) reviewed and edited the content as needed and take(s) full responsibility for the content of the publication.

## STAR★Methods

### Key resources table

**Table:** 

REAGENT or RESOURCE	SOURCE	IDENTIFIER
**Antibodies**		

CD19 FITC	BD Pharmigen	Cat# 561740
CXCR2 PE	R&D	Cat# FAB2164P
CD8a V500	BD Horizon	Cat# 560776
CD4 PerCP-Cy5.5	BioLegend	Cat# 100432
Ly6G APC	BD Pharmigen	Cat# 560599
APC-Cy7	BioLegend	Cat# 108724
CD192 (CCR2) BV421	BioLegend	Cat# 150605
Ly6C PerCP-Cy5.5	BioLegend	Cat# 128028
CD274 (PD-L1) PE-Cy7	BioLegend	Cat# 124313
CD184 (CXCR4) FITC	BD Pharmigen	Cat# 551967
CD11b APC-Cy7	BD Pharmigen	Cat# 557657
Ly6G BV421	BD Horizon	Cat# 562737
CD184 (CXCR4) BV421	BD Horizon	Cat# 562738
CD45 APC-Cy7	BioLegend	Cat# 103116
CD63 FITC	BioLegend	Cat# 143920
MPO	Thermo Fisher	Cat# PA5-16672

**Chemicals, peptides, and recombinant proteins**		

LIVE/DEAD™ Fixable Violet Dead Cell Stain Kit	Invitrogen	Cat# L34964
LIVE/DEAD™ Fixable Aqua Dead Cell Stain Kit	Invitrogen	Cat# L34966
Fixable Viability Dye eFluor™ 780	Invitrogen	Cat# 65-0865-18
Apotraker Tetra AF647	BioLegend	Cat#427406
Collagenase II	Worthington Biochemical	Cat# LS004177
INVANZ Ertapenem sódico	Merck Sharp & Dohme	Cat# 100026127048
Iscove’s Modified Dulbecco’s Medium	Corning	Cat# 10-016-CVR
Mueller-Hinton agar	BD Biosciences	Cat# 225250
Tween 20	Sigma-Aldrich	Cat# P9416
1X DPBS	Corning	Cat# 21-030-CVR

**Critical commercial assays**		

Next GEM Single Cell 3′ Kit v3.1	10x Genomics	Cat# 1000269
3′ CellPlex Kit	10x Genomics	Cat# 1000261
Quant-iT™ PicoGreen® kit	Thermo Fisher	Cat# P7589

**Deposited data**		

GEO dataset of raw and processed scRNAseq data	This paper	GEO: GSE287865

**Experimental models: Organisms/strains**		

C57BL/6 mice	The Jackson Laboratory	Cat# 000664
*Ifng*−/− mice	The Jackson Laboratory	Cat# 002287

**Software and algorithms**		

Prism 9	GraphPad	https://www.graphpad.com/updates/prism-900-release-notes
FlowJo v.10	BD Biosciences	https://www.flowjo.com
Cell Ranger	10X genomics	https://www.10xgenomics.com/support/software/cell-ranger/latest
R	The R Project for Statistical Computing	https://www.r-project.org
R studio	R studio	https://rstudio-education.github.io/hopr/starting.html

### Experimental model and study participant details

All mice (C57BL/6 reference number 000664 and *Ifng*^−/−^ reference number 002287) were purchased from The Jackson Laboratory and bred in the SPF animal facility of the Ribeirao Preto Medical School, University of Sao Paulo, Brazil. All experiments were conducted with 7-week-old male mice, weighing 20–25 g, and co-housed for one month to standardize the microbiota. All experiments and mice maintenance were approved by the Institutional Animal Care and Use Committee of the Ribeirao Preto Medical School (registration number 151/2019) and adhered to guidelines of the Care and Use of Laboratory Animals.^48^

### Method details

#### Model of sepsis and experimental protocols

Sepsis was induced using the cecal ligation and puncture (CLP) model, as previously described.^49^ The mice were anesthetized by inhalation of 1.5% of isoflurane, and two punctures were made through the ligated cecum using an 18-gauge needle. After the surgery, 1 mL of saline and tramadol 25 mg/kg was injected subcutaneously. After 6 h of the surgery, antibiotic treatment with 30 mg/kg ertapenem (Merck Sharp & Dohme) was administered, followed by six additional doses every 12 h. The survival rate of the mice was registered daily up to 7 days after CLP-sepsis induction. The blood samples were collected from the retro-orbital plexus of anesthetized mice (inhalation of 1.5% isoflurane). For tissue samples evaluation, mice were euthanized 24 h after CLP-sepsis induction by intramuscular injection of a lethal dose of a mixed solution of ketamine (100 mg/kg) and xylazine (10 mg/kg) in saline. Tissue samples were collected after transcardial perfusion with ice-cold 0.025% (wt/vol) heparin in PBS 1X. For cytokine determination, tissue were macerated, and the supernatants were collected. For flow cytometry analysis, the lung tissues were collected in Iscove’s Modified Dulbecco’s Medium (Corning, Cat#10-016-CVR) cut into small pieces with scissors, and digested for 45 min at 37°C with 1 mg/mL Collagenase II (Worthington Biochemical, cat# LS004177) in Iscove’s Modified Dulbecco’s Medium (Corning, Cat#10-016-CVR). After digestion, the tissues were gently pressed through a 70 μm nylon mesh cell strainer, and the resulting single-cell suspension samples were processed for flow cytometry. The peritoneal lavage samples were obtained by intraperitoneal infusion with 1.5 mL of ice-cold 1X DPBS (Corning, cat#21-030-CVR).

#### Bacteria quantification

Blood and peritoneal lavage samples were plated on Mueller-Hinton agar plates (BD Biosciences cat#225250), and the number of colony-forming units (CFU) was determined after 18 h of incubation at 37°C.

#### Measurement of organ damage biomarkers

The activities of alanine aminotransferase (ALT) and blood urea nitrogen (BUN) in plasma were measured according to the manufacturer’s instructions (Labtest, Brazil).

#### Cytokine assays

The concentrations of IL-6, IL-10, TNF-alpha, CCL2, CXCL1, CXCL2, and IFN-gamma in peritoneal lavage, tissue homogenate, and plasma samples were determined by ELISA following the manufacturer’s instructions (R&D Systems). The optical density of the individual samples was measured at 450 nm using a spectrophotometer (Spectra Max-250, Molecular Devices). Cytokine concentrations in tissue samples were normalized to the total protein concentration, which was determined using a bicinchoninic acid assay.

#### NETs quantification

The concentration of NETs was determined by detecting cf-DNA bound to MPO, as previously described.^47^ A 96-well clear-bottom black plate was coated with 50 μL of 5 μg/mL anti-MPO antibody (Thermo Fisher, cat#PA5-16672) for 16 h at 4°C. The plate was then washed three times with 0.5% Tween 20 (Sigma-Aldrich, cat#P9416) diluted in 1X PBS and incubated with 100 μL of 2% BSA diluted in 1X DPBS (Corning, cat#21-030-CVR) for 2 h at room temperature. After washing, 50 μL of plasma or cell culture supernatant samples and the concentration standard curve were added to the plate. After 16 h at 4°C, the plate was washed again, and the amount of the DNA was quantified using the Quant-iT PicoGreen kit following the manufacturer’s instructions (Thermo Fisher, cat#P7589). The fluorescence of the individual samples was measured at 488 nm of excitation and 525 nm of detection using a spectrophotometer (FlexStation 3, Molecular Devices).

#### Neutrophil cell culture

Naive mice were euthanized by intramuscular injection of a lethal dose of a with a mixed solution of ketamine (100 mg/kg) and xylazine (10 mg/kg) in saline. Femurs and tibias were then collected, and the cells were flushed with ice-cold 1X DPBS (Corning, cat#21-030-CVR). The cell suspension were gently pressed through a 70 μm nylon mesh cell strainer, and the resulting single-cell suspension samples were processed to the magnetic isolation according of manufactory’s instructions (Miltenyi Biotec Cat#130-097-658). The neutrophils were then resuspended in Iscove’s Modified Dulbecco’s Medium (Corning, Cat#10-016-CVR). Next, 25x10^4^ cells per well were plated in 96-well round bottom plates and incubated with media alone, 1000 ng/mL LPS (Sigma Aldrich, cat#L2630), or 10 ng/mL IFN-gamma (R&D, cat#485-MI) for 4 h at 37°C, 5% CO_2_ and 95% humidity.

#### Flow cytometry

Single-cell suspension from the blood, peritoneal lavage, and lungs were resuspended in ice-cold in-house prepared FACS buffer (2 mM EDTA, 25 mM HEPES, and 1% bovine serum albumin in 1× DPBS) and incubated with purified Rat Anti-mouse CD16/CD32 (BD Phamigen, cat#553141) for 10 min at room temperature. Extracellular markers were then stained with 1:300 dilution of fluorochrome-conjugated antibodies and 1:1000 dilution of viability dye for 15 min at room temperature. The list of fluorochrome-conjugated antibodies and viability dye is provided in the key resources table. Fluorescence and scatter signals were acquired by running samples on a BD FACSVerse (10 parameters) flow cytometer (BD Biosciences) and analyzed using FlowJo software version 10 (BD Biosciences).

#### Isolation of leukocytes and single-cell RNA sequencing

After 12 h of the CLP-sepsis induction, 50 μL of blood samples were collected from the retro-orbital plexus of anesthetized mice, and the sepsis survival rate was monitored for up to 7 days. Leukocytes were isolated using positive magnetic isolation of CD45^+^ cells, following the manufacturer’s instructions (Miltenyi Biotec, cat#130-052-301). Leukocytes from each mouse were the multiplexed with a molecular tag following the 10x Genomics 3′ CellPlex Kit (10x Genomics, cat#1000261), and cells from 10 mice were encapsulated in two lanes of a 10× Chromium instrument, each 5 mice per lane. Libraries were constructed using the Chromium Next GEM Single Cell 3′ Kit v3.1 following the manufacturer’s instructions (10x Genomics, cat#1000269), and were subsequently sequenced on the HiSeq 4000 (Illumina).

#### Single-cell RNA sequencing data pre-processing

Base call files were converted in the Cell Ranger to FASTQ files using the mkfastq function. The reads were then aligned to the mm10 *Mus musculus* transcriptome using the Cell Ranger software pipeline (version 6.1.2) provided by the 10x Genomics to generate raw genes by cell matrices of unique molecular identifier counts for each sample. The samples were then grouped in health controls, survived, and non-survived, and were read into R using the read10x function,^50^ resulting in a count matrix with 32285 genes and 8248 cells. A Seurat object was then created, and a filter was applied to remove the low-quality cells by excluding those based on unique molecular identifier counts (nCount_RNA <10000), the number of unique genes (nFeature_RNA between 200 and 3000), and the percentage of mitochondrial gene expression (percent.mt < 10). The remaining expression values were then normalized using the SCTransform (Hafemeister & Satija, 2019). The raw and processed data are available in GEO database identified as GSE287865.

#### Dimensionality reduction and clustering

The filtered and normalized matrix was used as input to the principal component analysis (PCA), and 40 components were selected for t-distributed stochastic neighbor embedding (UMAP) analysis and clustering. Data from the three groups were then integrated using the RunHarmony function from the Harmony package.^51^ Using the FindNeighbors and FindClusters functions, shared nearest neighbor clustering optimized with the Louvain algorithm resulted in cluster formation. The cluster annotation was performed using a combination of the scMRMA^52^ and scType^53^ packages, along with manual annotation based on gene expression data in the Cell Maker database.^54^

#### Differential expression

The differential expression testing was performed with Bonferroni correction using the FindMakers function. Only genes expressed in more than 10% of the compared cells and showing a minimum log[fold change] of 0.25 between groups were tested for significance.

#### Pathway enrichment

The set of differentially expressed genes (DEGs) (*p* value <0.05) for each comparison was used as input to determine significantly enriched Gene Ontology biological process and pathways by Fisher’s exact test using Enrichr (Kuleshov et al., 2016).

### Quantification and statistical analysis

Two-way analysis of variance (ANOVA) followed by Bonferroni was used to analyze multiple comparisons. The comparison between two groups was performed using Student’s t test. The statical analysis was conducted using Prism 9 (GraphPad Software), and the data are presented as mean ± s.e.m, each data point represents one mouse, and the statistical analysis results are demonstrated as *p* value in the figures. Differences that presented *p* values equal to or less than 0.05 were considered statistically significant.

### Additional resources

Our work is not part of, nor does it involve, a clinical trial. We disclose any clinical registry numbers and associated links.
