# Supplementary material for: CXCR4+ PD-L1+ neutrophils are increased in non-survived septic mice
Source: iScience. 2025 Feb 22;28(4):112083. doi: 10.1016/j.isci.2025.112083 (PMC12003019; doi:10.1016/j.isci.2025.112083)
Supplement: Document S1. Figure S1 and Tables S1–S3, S4, S6, S8, and S9 [file mmc1.pdf]

iScience, Volume *28*

Supplemental information

CXCR4^{+} PD-L1^{+} neutrophils are increased in non-survived septic mice

Guilherme Cesar Martelossi Cebinelli, Maísa de Oliveira Leandro, Antonio Edson Rocha Oliveira, Kalil Alves de Lima, Paula Barbim Donate, Cleyson da Cruz Oliveira Barros, Anderson dos Santos Ramos, Victor Costa, Daniele Carvalho Bernardo Nascimento, Luis Eduardo Alves Damasceno, Amanda Curto Tavares, André Nicolau Aquime Gonçalves, Helder Takashi Imoto Nakaya, Thiago Mattar Cunha, José Carlos Alves-Filho, and Fernando Queiroz Cunha

Supplementary Table 1: DEGs of CD4+ T lymphocytes between non-survived versus survived groups.

Genes	p_val	avg_log2FC
9930111J21Rik2	0.000945496509802149	-0.250637629212175
Ighm	0.000922862188473967	-0.257295247446919
mt-Nd4l	0.014013180734052	-0.257470632236534
Ablim1	0.00806478179536673	-0.258169497719382
Mycbp2	0.0027633926041946	-0.264042226371382
Arid1a	0.000642482189918748	-0.264949867194581
Bptf	0.00132120937315147	-0.265706184171216
Sell	0.00182367227376631	-0.275728610175005
Cd52	0.00146148950033568	-0.283752405174952
Cnn2	1,87E+09	-0.285729683319843
Gnai2	0.000174525185278095	-0.287444795518143
Rbm25	6,74E+09	-0.314806482003679
Sptbn1	0.000531112069151478	-0.322671661364154
Kmt2a	3,90E+06	-0.327450853036552
Ptprcap	0.000139636945871573	-0.330038198398542
Tmsb4x	2,89E+08	-0.349045811575205
Gm42418	6,54E+04	-0.472316184864743
Eif1	0.000878029532215974	0.250526019633466
Ifngr1	0.00549662741355127	0.251932173295378
Rps27	3,52E+05	0.260166279074381
Txnip	0.0150836606380587	0.272198390353497
Hif1a	0.00158107310760671	0.276233430014991
Rps21	1,22E+03	0.31615961442775
Hmgb2	0.00083907479784916	0.321341392166609
Trac	0.00318390318621345	0.328579761493878
Uba52	7,53E+08	0.363931333594305
H3f3b	6,47E+07	0.372843178275608
Gm10076	4,66E+08	0.398150470917448
Hbb-bs	5,94E+08	127.826.294.372.709

Supplementary Table 2: DEGs of CD4+ T naive lymphocytes between non-survived versus survived groups.

Genes	p_val	avg_log2FC
Hbb-bs	1,32E+07	228.699.293.240.425
Rpl34	3,90E+07	0.306206093574826
Zfp638	1,48E+07	-0.393821013369148
Gm42418	1,50E+08	-0.471155488954055
Rnf111	2,34E+08	-0.298720311158575
Tra2a	2,32E+09	-0.38455809204018
Lnpep	2,71E+09	-0.411379384753453
Rpl39	3,35E+09	0.281930154309125
Itga4	3,48E+09	-0.462945374023968
Macf1	3,93E+09	-0.518527262654969
Rps28	0.000115476824794213	0.319484077149396
Plec	0.00021178250673825	-0.350965534407351
Eif3f	0.000236867759240217	0.343144580797058
Actb	0.000258582519530898	-0.365053709190348
Cpsf6	0.000393321682348125	-0.290727520405045
Kif21b	0.000425048847461391	-0.306912948145983
Setd5	0.000438243321683705	-0.266065466170775
Pim1	0.000462018514071335	-0.395657671848765
Nsd1	0.000526363701613409	-0.318203966724453
Hbb-bt	0.000535311102919768	0.45517119853117
Sugt1	0.000543760929497944	-0.308932115782635
Rpl36a	0.000692967464995628	0.25645484660176
Srsf1	0.000696392330580942	-0.258028147105357
Ptprcap	0.000825506854733165	-0.377077749534572
H3f3b	0.000888338470002065	0.385744732204082
Pak2	0.00096507479366765	-0.302958214738002
Atf7ip	0.00116544643910521	-0.290222551887152
Dnaja1	0.00118351177040938	-0.278343795949212
Dgka	0.0015289558369956	-0.305690103090483
Tmsb4x	0.00171385155055994	-0.260571879989461
Ep400	0.00227327337400376	-0.257247960493704
Ssh2	0.00232864224390236	0.324648237179669
Sell	0.00244271823301649	-0.33071096951451
S100a10	0.00348863976525488	-0.311865417452355
Csde1	0.003671752302098	-0.254255023162321
Btla	0.00398973395027744	-0.258771053205295

Hnrnpul2	0.00400558464015115	-0.256102145930346
Ankrd44	0.0046947150248218	-0.25530114928772
Herc1	0.00473585210737158	-0.252465164123606
Rasa3	0.00486746634735733	-0.275501046180386
Gm10076	0.00543136480833372	0.343144580797058
Ubb	0.00564788316814887	0.287291346063541
Trim12a	0.00607838377040905	-0.350752291477263
Hba-a1	0.00636280120858775	117.993.292.344.118
Ltb	0.00638514608260625	-0.265025311085423
Atp1a1	0.00656001423909166	-0.31677997760532
Arhgap45	0.00687765021371231	-0.293745711967959
Chd4	0.00774149699823031	-0.277921893609346
Lars2	0.00855153097274814	-0.359033533795953
Nufip2	0.00883438495401031	-0.281895359299433
Hmgb2	0.00939853967213188	0.298232158921484
Laptm5	0.00949570106209959	-0.27869392215913
St8sia4	0.00950634638135785	-0.253890752224673
Cd52	0.00984032338992383	-0.343769201067386
Irf1	0.0102038880173523	0.308711083733184
Fam169b	0.010685502442648	-0.252958477529298
Nipbl	0.0108175330394519	-0.274607855041767
Pdia3	0.0113529530892588	-0.252159864145557
Smc4	0.011895858275682	-0.250329419779615
Kdm5a	0.0120368412126392	-0.304339004482157
Ripor2	0.0127223173149066	-0.278343795949212
Ywhab	0.0128508340965979	-0.258771053205295
Rac2	0.0132088090389949	-0.252958477529298
Hnrnpu	0.013939678137622	-0.293689554122841
Slc25a36	0.0156082129384177	-0.253313558761927
Il7r	0.0171780668802458	-0.286743790805614
Slfn8	0.0178012169020023	0.257104749730367
Txnip	0.0186646291041017	0.292833802664833
Mycbp2	0.0206243298173504	-0.263512991023417
Vps37b	0.0213106265505238	0.293176121522974
Ifit3	0.0214690299697748	0.277055390339286
Ncl	0.0218704282846566	-0.278068446193459
Cd28	0.0230010934107712	-0.255339059577717
Add3	0.0248419741396167	-0.286743790805614
Fam107b	0.0267427033825997	0.261401431355522

Top2b	0.0276633229792635	-0.313202553160863
Ablim1	0.0303583398565132	-0.268387640328001
Hba-a2	0.0378789244142068	124.005.108.783.295
S100a8	0.0401384438663307	-0.389862999800465

Supplementary Table 3: DEGs of CD8+ T lymphocytes between non-survived versus survived groups.

Genes	p_val	avg_log2FC
Trim12c	0.00075384464770127	-0.250606943911163
Igtp	0.00934803691205631	-0.251461718474443
Strn3	4,89E+08	-0.252246664478709
D8Ertd738e	0.000897115197236862	-0.253856815361123
Cd52	0.0265710757296566	-0.262746732849152
Igkc	2,58E+07	-0.263798950123488
Gbp9	6,34E+09	-0.26973794612987
Gbp2	0.00152467046983085	-0.27292124328901
Gimap4	0.00428035687280148	-0.274930615350193
Tmsb4x	0.00424322401489321	-0.280318381058474
Shisa5	0.00143980063763637	-0.283354763208628
Ccnd2	0.0127116060322449	-0.299736265095137
Sfr1	3,35E+09	-0.30050735454991
Ifi27l2a	0.0152398133800944	-0.301766800322508
Add3	9,23E+09	-0.304392269729332
Snrpd3	0.000929855448030331	-0.310034416306109
Actb	0.0079591687237704	-0.311560270537069
Heca	5,00E+09	-0.316467849668937
Calm1	0.00528106552276868	-0.321804553559054
Snrnp70	8,31E+09	-0.322525360489306
Dynll1	8,05E+09	-0.323100942001384
Pfn1	0.00828790382514245	-0.3263089845853
Ltb	7,17E+09	-0.329364358886538
Stat1	0.000794160346829678	-0.332754492806192
Hspa8	0.00059117936141424	-0.346056076467305
Lars2	3,94E+09	-0.364462059535933
Psme2	8,01E+09	-0.36960944610356
Gm42418	8,77E+06	-0.50406481624451
Txnip	0.00629631537687464	0.254274091096138
Tut4	0.0139483460035911	0.254851074704686
Trac	0.00999107785595147	0.261534917544055
Rps27	5,72E+07	0.267968894132538
Vps37b	0.00657757459748433	0.26889216236915
Ifngr1	0.00815133159387963	0.27028821661926
Rpl38	2,51E+07	0.273651927727777
Arid5a	0.0052506960337562	0.279507513368534

Ubc	8,66E+09	0.329131984565507
Hmgb2	5,83E+09	0.394227012787392
Hbb-bs	2,80E+08	0.420699224148583
Ubb	1,38E+06	0.460206362399074
Gm10076	5,96E+05	0.480568346878028

Supplementary Table 4: DEGs of CD8+ T ISGs lymphocytes between non-survived versus survived groups.

Gene	p_val	avg_log2FC
Ucp2	0.00331899247273857	-0.25541745342522
Trim12c	0.00539149607692982	-0.257113174430825
Stat1	0.00791760509883183	-0.258525197312071
Psmb10	0.00094640930662402	-0.262386688202687
AW112010	0.020596907999612	-0.271939959055686
Phf11b	0.00171536600303043	-0.285542747950551
Gbp7	0.000385883028781862	-0.292058226768279
Psmb8	7,84E+09	-0.307485484475414
Bst2	0.00034996193900051	-0.308346223081331
Ltb	0.00169636624254307	-0.313349849283808
Sell	5,56E+08	-0.319284401649632
Ifi27l2a	0.000100226240620658	-0.367975780557562
Tmsb4x	9,39E+07	-0.368353660640294
Cd52	0.000599099940158685	-0.369515023739693
Ifi203	1,03E+09	-0.405140120678804
Arap2	0.00372708504875346	0.255420853125335
Actg1	0.0161873872355127	0.267322310351623
Hif1a	9,15E+09	0.306194083501701
Ifngr1	0.000618468501420917	0.319156694784794
Zeb1	6,37E+08	0.321740516707686
Vps37b	0.00072398327339594	0.323477411198496
Gramd3	1,72E+09	0.328470508233573
Crybg1	4,01E+08	0.381732983454039

Supplementary Table 6: DEGs of B lymphocytes between non-survived versus survived groups.

Genes	p_val	avg_log2FC
Hbb-bs	1,84E+08	0.939409039269314
Hba-a1	0.000114713018370958	0.690657520814324
Klf13	1,40E+07	0.341123984823651
Txnip	0.00773117127468125	0.326747251907511
Gm10076	3,67E+07	0.32579860077322
Hnrnpdl	4,29E+09	0.309141599174316
Fam107b	9,19E+09	0.302993494860005
Hmgb2	0.00214266839693779	0.296798236782662
Sf3b6	7,75E+09	0.277986023416297
Socs3	3,59E+09	0.276323567153203
Kras	0.0078027149272327	0.274578429966787
Ptma	9,09E+08	0.258988302652657
Jun	0.00941249677627836	0.251584275072087
Ms4a4c	1,36E+00	-0.580389448254488
Sell	3,10E+01	-0.494742537511805
Ifi203	1,77E+03	-0.484379667531704
Fcmr	6,15E+04	-0.410069970268963
Ifi27l2a	9,61E+04	-0.406794838236102
Iglc2	2,80E+07	-0.395749295116936
Ly6a	2,59E+07	-0.377374033327039
Rnf213	3,63E+08	-0.375257945151591
Iglc3	1,52E+04	-0.36915369841113
Gm42418	4,61E+06	-0.335943774049872
Ltb	2,10E+06	-0.33153057383774
Igkc	3,41E+08	-0.331303176053022
Serinc3	1,75E+08	-0.324241512218836
Macf1	2,01E+09	-0.318101136345733
Ighd	7,49E+08	-0.311515388249152
Ifi209	4,46E+08	-0.310602607759558
Isg15	0.000362546166625878	-0.309647194039364
Slfn5	0.00268296797389064	-0.307575907367979
Phf11b	1,09E+08	-0.30574459321804
Irf2	1,41E+08	-0.29840174156685
Rnase6	1,37E+07	-0.298329853033019
Crlf3	3,07E+08	-0.294632009322843
Pou2f2	0.000104344891782757	-0.292860946989896

Cst3	5,21E+08	-0.292103675433019
H2-T23	0.000169900223814622	-0.291812323097282
Bst2	0.000130242539110936	-0.28579659669629
Smim14	1,46E+07	-0.282516227736008
BE692007	6,70E+09	-0.271702190671567
Ly86	0.00054733054951031	-0.271018547196028
H2-K1	6,23E+07	-0.270886987593889
Trim30a	4,82E+09	-0.269646395718796
Ifi214	2,05E+09	-0.269533043132536
Cd79b	4,45E+08	-0.263618436558006
Herc6	1,53E+07	-0.26234771923941
Shisa5	3,57E+09	-0.261883492845709
Kmt2a	3,73E+08	-0.261130949604357
Ifit2	0.00244636653213163	-0.259655677237867
Fchsd2	9,37E+08	-0.257617788869816

Supplementary Table 8: DEGs of mature neutrophils between non-survived versus survived groups.

Genes	p_val	avg_log2FC
Spn	0.00405807847612228	-0.251379415374826
Myo1f	0.00073936429951487	-0.252485200779503
Cdc42se1	0.00301698291202966	-0.253513868819275
Ehd1	0.0118627423120122	-0.260060054588197
Dusp1	0.0103162292041891	-0.260253557745937
Cxcr2	0.0131083200260249	-0.26044247872321
Igkc	0.00247340644922148	-0.260878140825537
Ifit3b	0.00199522016461934	-0.26182355045366
Gm31814	0.130047375266289	-0.262720170206014
Marcks	0.00180834001591639	-0.265486137317029
Rin3	8,86E+09	-0.269431261950465
Arhgap30	0.000529775714405633	-0.271905258211307
Fgr	2,51E+09	-0.275441108812047
Capzb	0.000733350784730151	-0.277129419917608
Emp3	0.0454260220259592	-0.278030230205896
Lbr	0.00272216017064877	-0.278279163868991
Gpsm3	4,16E+09	-0.279500579243956
Itgb2	0.00485136734108129	-0.279744189344845
G0s2	0.0556582713802288	-0.28095680749903
Nadk	0.000272172719500438	-0.284148909650816
Gda	0.000158773887234452	-0.290978283693353
Pik3cd	8,93E+09	-0.291658193670191
Cst3	0.000159879342612997	-0.292402810482018
Sp100	2,08E+09	-0.294825472748874
Slc16a6	0.00198062720593678	-0.295219597882015
Lars2	0.00464211514996507	-0.297465354621578
Trim30d	6,57E+09	-0.30098599582531
Ankrd44	0.00118447234007174	-0.303093088732474
Mob1a	9,91E+08	-0.303673922817039
Ifitm1	0.000207340321401134	-0.306186967422774
Stfa2l1	0.180067103305309	-0.307357573772314
Gm19951	5,45E+09	-0.307764528456372
Ifi204	0.000582324594785003	-0.308933692365335
Trim30b	0.00130574470421174	-0.309325042443989
Fgd4	2,02E+08	-0.311327395355137
Efhd2	0.000830095231450924	-0.312806948245098

Trim30a	0.000561674095966415	-0.31354323934788
Btg2	0.000107363872702288	-0.314054944228927
Pgd	0.000113712526265574	-0.322896238335542
Il1b	0.00284889984128979	-0.32577476159351
Adgre5	0.000504056425502714	-0.328493185514269
Tsc22d3	1,63E+09	-0.332054397512378
Gpcpd1	5,37E+09	-0.335177371373755
Itgal	0.000617190795025591	-0.34459511957474
Irf7	7,07E+09	-0.355774369398101
Ifi27l2a	0.0238859947212362	-0.364286574749548
Selplg	1,02E+08	-0.370731805607838
Prok2	0.00508165429936116	-0.371202870479869
Slfn4	5,17E+09	-0.373184554531168
Csf3r	0.00175912030952858	-0.391970254378124
Gm42418	1,17E+05	-0.404701746252865
Marcksl1	0.00266198693440996	-0.405461427099311
Neat1	0.000176213733639484	-0.41039641731134
Lst1	4,22E+09	-0.413382083100782
Saa3	0.029749091879291	-0.432934436456084
Stk10	2,35E+08	-0.441630867937973
Pglyrp1	1,40E+08	-0.472234407714979
Prr13	5,56E+06	-0.474018012136899
Gngt2	0.00065411285982427	-0.485938727597464
Rgs3	3,99E+06	-0.497707070247879
Pou2f2	1,89E+09	-0.502393246641899
Ltb	3,75E+07	-0.502645092998049
Gpx1	1,89E+06	-0.532577530094931
S100a8	0.00771582244342431	0.25116891534489
Rps13	0.569817739586221	0.25141165870052
Rpl19	0.437332945275502	0.254005082303257
Jun	0.0586670169813362	0.258780431716573
Srsf5	0.0020049406908201	0.261012475453815
Rab8b	0.000585698576684779	0.261737368039977
Hbb-bt	0.00131119255397036	0.262093990790479
Rps5	0.429016022814252	0.264808355845235
Lmnb1	0.00608639200636589	0.267342445219997
Gm10076	0.00275809229769272	0.271913269353375
Eef1a1	0.324249953051398	0.274039845175348
Pik3ap1	0.000755879377863837	0.27963148093871

Rapgef6	0.0333758373557816	0.281424596635709
Emb	0.0169199779200049	0.281483716212855
mt-Atp6	0.0241645089570376	0.282104447140607
Asprv1	0.72903542985483	0.283682005834983
Tnfaip3	0.00171141861154629	0.300304054129281
Mt1	0.524823447269518	0.306670960056135
Esd	0.0824354915140171	0.308369713480052
Hdc	0.0247209450932053	0.316996834833289
Rps28	0.51778537223526	0.318562538997914
Rplp0	0.132326676995687	0.318860715945209
Fth1	0.0748058482786132	0.320336338780842
Rps27	0.0703828796209765	0.323266887550297
H3f3b	3,98E+08	0.324785256275177
Gns	0.000109811437529472	0.325240828928198
Cstdc4	0.315399650639497	0.326252697841408
Hcar2	0.000223639231296932	0.335062898853799
Ier3	0.005998548021039	0.347470203038234
Ccrl2	0.00259397322084003	0.362079620048539
Rpl12	0.0847758429566267	0.375501199831217
Nfkbia	0.000133522190648648	0.395823344212868
Hba-a1	0.08831157737752	0.401862110531111
Hba-a2	0.0284647588511257	0.408192789493994
Camp	0.0657271446535965	0.42339647320307
Cstb	0.0217598351254536	0.434078914338997
Ltf	0.146153141642118	0.472337209317337
Hif1a	1,82E+06	0.472489350906844
mt-Nd1	0.00344426547096699	0.489151630790405
Thbs1	0.000142090460509222	0.511221291439509
Ngp	0.00520798986086945	0.516700519217324
Ccl3	0.00252490780518578	0.541675794968246
Cxcl2	0.0127771171829853	0.566776928569814
Cxcl3	0.00210125448607263	0.676642426136614
Hbb-bs	3,54E+08	0.696105018005279
Ccl4	0.00149874593830392	0.94838395472867

Supplementary Table 9: DEGs of immature neutrophils between non-survived versus survived groups.

Genes	p_val	avg_log2FC
Gbp2	5,97E+08	-0.251238008787322
Neat1	3,34E+09	-0.2529757691292
Hspa5	0.00894842689996495	-0.253246621954969
Cd52	1,03E+07	-0.256778602312929
Rplp1	1,81E+08	-0.258629917078806
Vim	8,52E+08	-0.260135776712292
Ctss	1,22E+07	-0.260604351545373
Cmah	7,46E+07	-0.261805424792544
Rsrp1	7,69E+05	-0.264076113119221
Apobec3	1,35E+09	-0.264410362602464
Nfam1	2,68E+09	-0.265435094983921
Marcks	2,77E+09	-0.26581067805264
Crispld2	8,56E+08	-0.267260395835686
Msrb1	8,61E+06	-0.267637437234395
Ddx60	8,66E+09	-0.270431959729288
Tgfbi	1,29E+08	-0.271795944909312
Taldo1	8,25E+08	-0.276714311980937
Fabp5	1,47E+08	-0.279720173030694
Stfa2l1	0.00256630066718757	-0.280840527476775
Ifi204	1,21E+09	-0.284658095846361
Pgd	1,03E+08	-0.287053494490568
Samhd1	1,39E+08	-0.287960862991365
Ly6e	2,40E+09	-0.291915109542454
Isg20	2,00E+09	-0.296262987178121
Baz1a	2,31E+07	-0.297684270904492
Ly6c2	3,28E+09	-0.297971591991461
Rpl12	4,57E+07	-0.298463895371392
Oasl2	2,89E+08	-0.304505702159225
Mmp8	3,51E+08	-0.308783557452743
Irf7	4,18E+08	-0.317779359517003
Cd44	4,67E+05	-0.327225190974642
Sell	3,61E+07	-0.327610241030908
Herc6	9,75E+06	-0.327982982291447
Prr13	3,72E+06	-0.32860566756532
Malat1	6,06E+02	-0.332031918050083
Mmp9	3,38E+03	-0.348333144795996

Gda	7,88E+05	-0.357109036738776
Ifit3	4,78E+09	-0.360886395905207
Cxcr2	1,77E+06	-0.361093525332405
Lipg	7,24E+05	-0.361127930153562
Saa3	0.00602358982864976	-0.3678230946643
Rsad2	0.000542453409255523	-0.374915955809265
Gm42418	1,12E+01	-0.377566645467423
Slfn4	1,34E+06	-0.416257790437455
Prok2	8,26E+08	-0.418045511124064
Ifit1	2,24E+09	-0.427577273296716
Slfn5	1,07E+08	-0.449571738995043
Isg15	9,86E+07	-0.451688059596817
Gpx1	8,69E+02	-0.465190640865421
Ifitm1	0.00149833165845887	-0.574752115105049
Wfdc17	3,85E+04	-0.617933199649296
Mt1	0.000782044478389596	0.25323072397169
Map3k8	3,16E+06	0.256925489966417
H2afz	0.000195622702812875	0.259049085619913
Ets2	2,89E+08	0.267366745171568
Ltf	1,73E+08	0.27511384187014
Plscr1	0.00010619448121698	0.282354550940217
Orm1	0.000113213722325466	0.318770424088768
Ngp	6,50E+00	0.322611825812724
H3f3b	2,42E+03	0.330709498853179
Rasa2	1,06E+07	0.339418919625547
Nfkbia	1,04E+09	0.364246743714174
Il1r2	4,12E+04	0.36477623674599
Samsn1	1,03E+05	0.381190780103937
Cd14	0.000213023348560492	0.382648464061297
Camp	1,50E+06	0.436434225340605
Hbb-bs	1,67E+04	0.479985150239835
Thbs1	1,37E-04	0.813009351479649

Figure S5 – Evaluation of the sepsis parameters in mice predicted to survive and non-survive after CLP-sepsis. a, Receiver Operating Characteristic (ROC) analysis of IL-6 concentration at 24 h after CLP-sepsis induction and the outcome assessed until 7 days (survived n = 8 and non-survived n = 14). The plasmatic IL-6 concentrations higher than 4165 pg/mL had 100 % specificity to identify mice non-survived, and the plasmatic IL-6 concentrations lower than 2790 pg/mL had 100 % specificity to identify mice survived. **b,** The plasmatic concentration of IL-6 in controls, mice predicted to survive and non-survive after 24 hours of the CLP-sepsis induction. **c,** The bacteria quantification and cytokines concentration in the **d,** peritoneal cavity, and **e,** the lungs, liver, heart, and kidneys of controls, mice predicted to survive and non-survive after 24 hours of the CLP-sepsis induction. The data is representative of two independent experiments with similar results. The data are shown as means ± s.e.m and each data point represents one mouse. Statistical significance was determined by one-way ANOVA followed by Tukey's multiple comparisons test.

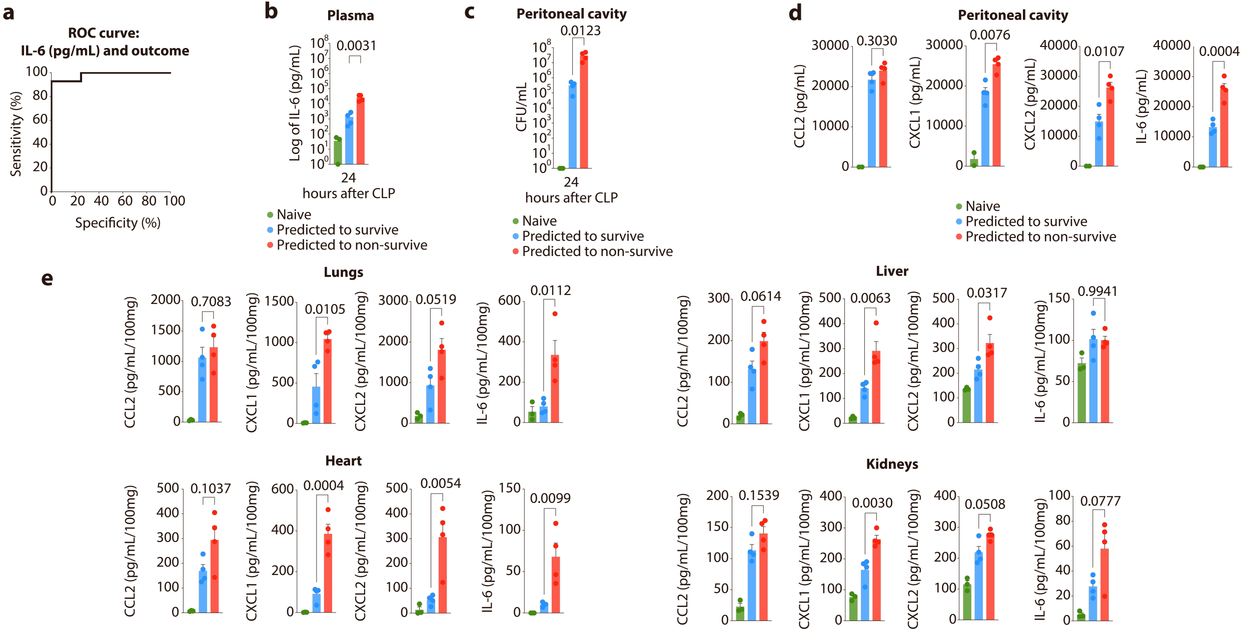

a
ROC curve:
IL-6 (pg/mL) and outcome
Sensitivity (%)
Specificity (%)
b
Plasma
Log of IL-6 (pg/mL)
0.0031
24
hours after CLP
Naive
Predicted to survive
Predicted to non-survive
c
Peritoneal cavity
CFU/mL
0.0123
24
hours after CLP
Naive
Predicted to survive
Predicted to non-survive
d
Peritoneal cavity
CCL2 (pg/mL)
0.3030
CXCL1 (pg/mL)
0.0076
CXCL2 (pg/mL)
0.0107
IL-6 (pg/mL)
0.0004
Naive
Predicted to survive
Predicted to non-survive
e
Lungs
CCL2 (pg/mL/100mg)
0.7083
CXCL1 (pg/mL/100mg)
0.0105
CXCL2 (pg/mL/100mg)
0.0519
IL-6 (pg/mL/100mg)
0.0112
Liver
CCL2 (pg/mL/100mg)
0.0614
CXCL1 (pg/mL/100mg)
0.0063
CXCL2 (pg/mL/100mg)
0.0317
IL-6 (pg/mL/100mg)
0.9941
Heart
CCL2 (pg/mL/100mg)
0.1037
CXCL1 (pg/mL/100mg)
0.0004
CXCL2 (pg/mL/100mg)
0.0054
IL-6 (pg/mL/100mg)
0.0099
Kidneys
CCL2 (pg/mL/100mg)
0.1539
CXCL1 (pg/mL/100mg)
0.0030
CXCL2 (pg/mL/100mg)
0.0508
IL-6 (pg/mL/100mg)
0.0777
